# Lifestyle Behaviors and Cognitive Well-Being: A Cross-Sectional Study Exploring the Role of Lifestyle Factors Among Omani University Students

**DOI:** 10.3390/ijerph23010017

**Published:** 2025-12-22

**Authors:** Maha AlRiyami, Amal Saki Malehi, Fatema Al-Mazidi, Almundhir Humaid Alomairi, Zakriya Nasser Al-Manji, Arwa Al Kindi, Helia Bolourkesh, Siham Al Shamli, Alya ALBusaidi, Samir Al-Adawi

**Affiliations:** 1 Department of Biochemistry, College of Medicine and Health Sciences, Sultan Qaboos University, Muscat 123, Oman; arwank@squ.edu.om; 2 Family Medicine and Public Health, College of Medicine and Health Sciences, Sultan Qaboos University, Muscat 123, Oman; a.malehi@squ.edu.om; 3 College of Medicine and Health Sciences, Sultan Qaboos University, Muscat 123, Oman; s136468@student.squ.edu.om (F.A.-M.); s138010@student.squ.edu.om (A.H.A.); s136615@student.squ.edu.om (Z.N.A.-M.); 4 Department of Behavioral Medicine, College of Medicine and Health Sciences, Sultan Qaboos University, Muscat 123, Oman; heliablk21@gmail.com (H.B.); adawi@squ.edu.om (S.A.-A.); 5 Department of Behavioral Medicine, Sultan Qaboos University Hospital, University Medical City, Muscat 124, Oman; s.alshamli@squ.edu.om; 6 Medical City Hospital for Military and Security Services, Muscat 124, Oman; alya.b19@resident.omsb.org

**Keywords:** subjective cognitive well-being, physical activity, chronotype, disordered eating attitudes and behaviors, university students, lifestyle behaviors

## Abstract

Poor coping among university students is widespread globally, yet few studies examine whether modifiable lifestyle risk factors are associated with this phenomenon. This study aims to assess the frequency of physical activity, chronotype, and disordered eating attitudes among students, and to determine whether these factors are associated with effective functioning in academic settings and subjective cognitive well-being. A cross-sectional survey was conducted among Omani undergraduate students (*n* = 408) using a questionnaire covering sociodemographic characteristics and instruments, including the International Physical Activity Questionnaire, the Morningness–Eveningness Chronotype Scale, the Eating Attitudes Test, and a measure of subjective cognitive well-being. Participants’ mean age was 20.21 ± 1.57 years (female = 74.3%). In total, 28.4% showed disordered eating attitudes, and half were physically active. 34.1% were classified as evening type. Independent regression analysis showed that chronotype was positively associated with physical activity (β = 0.06, *p* = 0.004). Disordered eating behavior did not significantly associate with physical activity (β = 0.1, *p* = 0.16). Moreover, physical activity was positively associated with cognitive function (β = 0.11, *p* = 0.039). However, the effect sizes were small, suggesting additional factors may contribute to these associations. This study is among the first to explore the influence of lifestyle factors on cognitive well-being in university students and may inform future studies and interventions targeting modifiable lifestyle behaviors to improve coping and academic functioning.

## 1. Introduction

University students are increasingly vulnerable to chronic stress, leading to a spectrum of poor mental health outcomes and reduced coping capacity. According to the World Health Organization’s World Mental Health International College Student project, approximately 20.3% of college students report having mental health-related disorders [1]. Moreover, the COVID-19 pandemic has been shown to have a significant negative impact on long-term health and quality of life among young adults. Persistent cognitive, physical, and mood-related impairments are common after the acute phase. They are further influenced by lifestyle factors such as physical inactivity, smoking, sleep patterns, diet, and overall health behaviors [2].

In a country such as Oman, where the majority of its population is engaged in school or tertiary education, scattered data indicate that the educational setting is not immune to the vagaries of chronic stress [3]. With the increasing burden of such tribulation in Oman and in other countries, most efforts have focused on quantifying the extent of the problem. On the other hand, conventional approaches, such as talk therapy, stress management strategies, and pharmacological interventions, have demonstrated limited effectiveness [4]. As a result, many students continue to experience burnout, which can contribute to underachievement and higher dropout rates [5], ultimately leading to reduced occupational preparedness, diminished future work performance, increased healthcare utilization, and greater disability and dependency [6].

Given these concerns, there is an urgent need for a comprehensive shift in how we address the challenges students face in higher education. Lifestyle factors, long considered peripheral, may be key determinants of student well-being. In this context, the current study explores the role of three lifestyle behaviors: physical activity, sleep chronotype, and disordered eating attitudes and behaviors. These were previously described as “the big three health behaviors” [7] as they are foundational pillars associated with health, well-being, and quality of life. On one hand, when balanced, these three interrelated domains are believed to play a crucial role in supporting physical, emotional, and cognitive well-being; on the other hand, physical inactivity, poor sleep patterns, and disordered eating have been identified as precursors to burnout [8,9].

Understanding how variations in these lifestyle behaviors are associated with subjective cognitive status aligns with global public health efforts to integrate lifestyle interventions aimed at reducing the burden of metabolic and psychological distress, which are globally major public health priorities. Focusing efforts and policies on prevention is more efficient and cost-effective than treatment. This is particularly important among university students, as mental health issues contribute to economic and social costs through weakened academic performance, and increased healthcare demand and medication use [1]. Moreover, schools and universities are ideal environments for shaping healthy lifelong health habits and implementing public health policies targeting modifiable risk factors such as physical activity, eating behaviors, and chronotypes allows for early engagement of a large population with habits that can produce broad, multilevel benefits from personal to national scales [7]. In the following section, the three lifestyle behaviors are considered in tandem.

First, physical inactivity and sedentary behavior are widely recognized as modifiable risk factors associated with poor physical and mental well-being among university students [10,11]. Compared with the general population, higher education students report lower levels of physical activity, partly attributed to prolonged periods of study and screen time, and limited access to environments conducive to physical activity [12]. Although physical activity has been examined in the general population in Oman [13], there is a notable lack of research focusing specifically on tertiary education, and none has examined physical activity in relation to cognitive well-being.

Second, chronotype refers to an individual’s natural preference for the timing of sleep and wake within a 24 h period, influenced by underlying circadian rhythms [14]. Individuals may be categorized as morning ‘larks’, evening ‘owls’, or an intermediate type. These preferences are associated with cognitive performance, mood regulation, and social functioning [15,16]. Thus, chronotype may have implications for academic performance, particularly in educational environments that fail to accommodate circadian variability. Sleep deprivation and irregular sleep are associated with impaired cognitive functioning, emotional regulation, and stress management, potentially increasing vulnerability to cognitive decline. Despite its relevance, the role of chronotype in shaping the cognitive and academic functioning of university students remains underexplored, particularly in the Arabian Gulf region, and Oman is no exception.

Third, eating behaviors such as restricted eating, binge eating, compulsive eating, and irregular meal patterns are increasingly observed among college populations [17]. These disordered attitudes and behaviors, although differing in severity and expression, are widely prevalent, including in the Arabian Peninsula [18]. University students are particularly at risk due to transitional stress, body image concerns, and irregular daily routines [19]. Irregular or unhealthy eating patterns can affect both physical and mental health, contributing to energy deficits, mood instability, and reduced coping capacity. However, little research has examined how disordered eating is associated with students’ cognitive well-being among students in tertiary education.

The interrelationship between physical inactivity, poor sleep patterns, and disordered eating is still poorly understood, as previous studies have examined the associations of each factor with mental health and cognitive well-being independently. This study offers a novel approach by investigating the combined associations of the three factors with cognitive well-being, aiming to develop a hypothetical model of potential pathways, rather than examining each factor separately. This approach may inform future policies by encouraging a shift from single-factor interventions toward integrated, multifactor strategies that promote cognitive well-being and foster a healthier campus environment that supports students’ holistic well-being.

This study aims to assess the frequency of these lifestyle behaviors and to examine whether variations in physical activity, sleep chronotype, and disordered eating attitudes are associated with subjective cognitive status among undergraduate students. It is hypothesized that misaligned circadian rhythm, insufficient physical activity, and disordered eating habits are associated with the cognitive functioning of tertiary education students. By addressing this gap, this study aims to contribute to a growing body of evidence supporting holistic, lifestyle-based interventions that promote academic and psychosocial well-being.

## 2. Materials and Methods

### 2.1. Ethical Consideration

This study was approved by the Medical Research Ethics Committee (MREC) of the College of Medicine and Health Sciences at Sultan Qaboos University (Ref. No. 3447, 25 November 2024). Informed consent was obtained from all study participants.

### 2.2. Aims and Study Design and Setting

This study aims to explore the trajectories of three lifestyle behaviors: physical activity, sleep chronotype, and eating attitudes, in relation to cognitive well-being. A cross-sectional study was conducted among undergraduate students at Sultan Qaboos University (SQU), located on the outskirts of Muscat, the capital city of Oman. As of 2024, SQU enrolled approximately 15,852 students from diverse geographical and socioeconomic backgrounds across the country [20,21]. The university offers a broad range of undergraduate programs in the sciences, humanities, and arts, as well as postgraduate programs, including master’s and doctoral degrees. Oman provides free universal education through the secondary level, and students seeking tertiary education typically compete for government scholarships to study at SQU.

### 2.3. Study Participants and Data Collection

All full-time undergraduate students enrolled at SQU were invited to participate. The questionnaire was distributed electronically using Google Forms. Previous studies have suggested that this approach is likely to capture a broad representations of the student body at the present setting [21]. The first page of the questionnaire included an informed consent statement outlining the study objectives, emphasizing voluntary participation, and ensuring confidentiality and anonymity. This approach reduces the likelihood of sampling bias. No personally identifiable information was collected. The survey included a statement advising participants to inform the principal investigator or to seek assistance from campus health or counseling services if participation triggered any adverse experience. Data were collected between 21 August 2024 and 1 November 2025.

### 2.4. Inclusion and Exclusion Criteria

Inclusion criteria included Omani, Arabic-speaking male and female full-time undergraduate students at SQU aged. Exclusion criteria included non-Arabic speaking students and those younger than 18 or older than 25 years.

### 2.5. Sample Size

Using a 95% confidence level and a 5% error margin, this sampling approach was deemed sufficient to represent the student population in this setting. Power analysis for this study was conducted using the Epi Info version 6.0 software. The sample size was estimated with a Type I error of 5% (alpha = 0.05) and a 95% confidence level to achieve 80% statistical power. Based on these parameters, a minimum of 375 undergraduate students was required to detect a 50% difference in the odds ratio (OR) between groups of interest. A total of 408 students aged 18–25 years participated in this study (females: *n* = 303; males: *n* = 105).

### 2.6. Sociodemographic Data and Risk Factors

Sociodemographic data and risk factors were collected using a self-administered questionnaire that captured age, sex, marital status, academic year, college and major, duration of university enrollment, academic performance, place of residence, type of accommodation, and level of parental education level. (See Table 1 for details).

Health and life background information was collected through self-report, including chronic health conditions, current medication use, family history of mental illness, smoking habits, and alcohol consumption. Participants were also asked whether they had experienced adverse childhood experiences. The adverse childhood experiences assessed in this study were those defined by the Centers for Disease Control and Prevention (CDC) [22].

Disordered eating attitudes and behaviors were assessed using the 26-item Eating Attitudes Test (EAT-26), developed by Garner and Garfinkel (1979) [23]. The EAT-26 has demonstrated robust psychometric properties in Arabic-speaking populations, including Oman [24]. A cutoff score of 20 was used to identify individuals at risk for disordered eating [23].

Physical activity was assessed using the Short Form of the International Physical Activity Questionnaire (IPAQ-SF) [25], a validated self-report instrument. The IPAQ-SF measures physical activity across four domains (vigorous activity, moderate activity, walking, and sitting time) during the preceding seven days. This study used the validated Arabic version of the IPAQ-SF [25,26]. Participants were classified as ‘active’ or ‘inactive’ according to the established scoring guidelines [25].

Chronotype was assessed using the 5-item Morningness–Eveningness Questionnaire (MEQ-5) (5 items) [27], a validated instrument for determining an individual’s circadian preference. Total scores range from 4 to 26, with higher scores indicating a morning chronotype. Classification followed the criteria of Adan and Almirall (1991) [28]: evening type (<12), neither type (12–17), and morning type (>17). This study used the validated Arabic version of the MEQ-5 reported by Hazmi and Noorwali (2023) [29].

Subjective cognitive well-being was assessed using an adaptation of the Questionnaire of Memory Efficiency (QME) [30]. QME was utilized in cognitive domains such as concentration, orientation, episodic memory, and prospective memory. Since no Arabic version is available, this study also translated QME into Arabic. Two board-certified neuropsychologists fluent in Arabic participated in translation to ensure conceptual and linguistic validity. The final version demonstrated adequate internal consistency (Cronbach’s alpha = 0.85).

The psychometric properties of the outcome measures were as follows: Cronbach’s alpha for sleep–wake chronotype = 0.71, disordered eating behavior = 0.83.

### 2.7. Statistical Analysis

Participants with missing data were excluded from the analyses. Categorical variables were reported as frequencies (percentages) for EAT-26, IPAQ and chronotype, and continuous variables were reported as means with standard deviations. The Kolmogorov–Smirnov test was used to assess the normality of continuous data. Associations between categorical covariates and disordered eating, physical activity, and chronotype were tested using the chi-square test. Individual linear regression analyses were conducted to examine the association among disordered eating (continues format), sleep–wake chronotype (continues format), and physical activity (categories), as well association between physical activity and subjective cognitive function. Structural equation modeling (SEM) was conducted to test a conceptual model hypothesizing associations between disordered eating, physical activity, and sleep–wake chronotype and subjective cognitive function. Direct, indirect, and total effects were calculated using the bootstrap method (5000 samples; 95% confidence interval). All analyses were performed with SPSS version 29 (IBM Corp., Armonk, NY, USA). SEM analysis was performed using SmartPLS (free trial version). A *p*-value < 0.05 was considered statistically significant for all analyses.

## 3. Results

### 3.1. Demographic Characteristics and Risk Factors of Participants

A total of 408 students participated in this study. As shown in Table 1, the mean age of participants was 20.21 ± 1.566 years. The majority of participants were females (*n* = 303, 74.3%), while males accounted for *n* = 105 (25.7%). Most participants were single (*n* = 397, 97.3%), and only 11 (*n* = 11, 2.7%) were married.

Regarding parental education, *n* = 285 (71.1%) fathers had university or institute-level education, while *n* = 99 (24.7%) had school-level education, and 4.2% were illiterate. Among mothers, *n* = 201 (49.3%) had a university degree, *n* = 161 (39.5%) had school-level education, and *n* = 44 (10.8%) were illiterate. In terms of housing arrangements, *n* = 221 (54.2%) participants resided on campus. Among students living off campus, most lived with their families (*n* = 116, 63.7%), followed by those in shared accommodation (*n* = 55, 30.2%). Academically, *n* = 202 (49.5%) students were rated ‘very good’, and *n* = 113 (27.7%) were rated ‘good’. Participants were distributed across various colleges, with the highest representation in the College of Medicine and Health Sciences (*n* = 131, 32.1%), followed by the College of Sciences (*n* = 62, 15.2%), and the College of Education (*n* = 43, 10.5%).

Most respondents did not report chronic disease, whereas *n* = 31 (7.6%) did. A small proportion of the participants (*n* = 39, 9.6%) had sought unified help for issues such as psychological problems. Similarly, *n* = 32 students (7.8%) reported having a history of adverse childhood experiences. Behavioral risk factors were minimal: only three students (0.7%) reported smoking, and none reported alcohol consumption, indicating a complete abstinence rate (100%) from alcohol in this group.

As shown in Table 2, *n* = 292 (71.6%) participants had eating attitude (EAT-26) score below 20, indicating no disordered eating attitudes, whereas *n* = 116 (28.4%) scored above 20, suggesting possible concerns about eating behavior. Regarding physical activity levels, *n* = 212 (52.3%) participants were classified as active, and *n* = 193 (47.7%) were classified as inactive. In terms of chronotype, *n* = 92 (22.5%) participants were identified as morning type, *n* = 139 (34.1%) as evening type, and *n* = 177 (43.4%) as neither type.

### 3.2. Exploratory Analysis

In this section, we conducted an exploratory analysis to investigate the associations between participant characteristics and the three study domains (Table 3). The result showed significant association between disordered eating behavior and seeking unified help for psychological or socioeconomic problems (*p* = 0.002), as well as a history of adverse childhood experiences (*p* = 0.023). Participants with chronic diseases requiring medical follow-up showed a significant association with disordered eating behavior (*p* = 0.039). However, no significant associations were obsereved for age, sex, marital status, accommodation, or smoking status (Table 3).

Additionally, there was a significant association between physical activity levels and participants’ sex (*p* = 0.009) and accommodation status (*p* = 0.017), with more females classified as physically inactive, 51.5% (155/301), compared with males, 36.5% (38/104). Students residing on campus were also more likely to be inactive (53.2%) than those living off campus (41.1%). For the sleep–wake chronotype, a significant association with academic level (*p* = 0.044) (Table 3).

### 3.3. Association with Subjective Cognitive Well-Being

The results of the regression analyses are shown in Table 4. In the first equation model, eating attitudes and chronotype were treated as independent variables, and physical activity was the dependent variable. In the second equation model, physical activity was the independent variable, and cognitive well-being was the dependent variable.

The first equation model showed that chronotype was positively associated with physical activity (β = 0.06, *p* = 0.004). However, disordered eating behavior was not significantly associated with physical activity (β = 0.1, *p* = 0.16). The second equation model showed that physical activity was positively associated with cognitive function (β = 0.11, *p* = 0.039). However, the effect sizes in both equations indicated small effect (β < 0.2) between the variables and the dependent variables.

SEM was implemented to examine potential chain-mediating effects among disordered eating behavior, chronotypes, physical activity, and subjective cognitive well-being among college students. We evaluated a range of possible models to identify the best and most appropriate model for assessing how physical activity, sleep–wake chronotypes, and disordered eating behaviors are associated with subjective cognitive well-being (Supplementary Figure S1). As shown in Supplementary Figure S1 and Table S1, chronotypes (β = 0.179, *p* = 0.001) and disordered eating behavior (β = 0.10, *p* = 0.047) were positively associated with physical activity, and physical activity was significantly associated with subjective cognitive well-being (β = 0.151, *p* = 0.005). However, the standardized βs were below 0.2, indicating small effect sizes. In addition, the total effect indicated that disordered eating behavior was not associated with subjective cognitive well-being (β = 0.015, *p* = 0.115). Chronotypes were significantly associated with subjective cognitive well-being (β = 0.027, *p* = 0.046) (Supplementary Table S1). The goodness-of-fit indices for the SEM model were as follows: standardized root mean squared residual (SRMR) = 0.29, normed fit index (NFI) = 0.65. This suboptimal fit could be due to the non-normal data distribution, or it may reflect potential involvement of additional unmeasured factors that warrant further investigation.

## 4. Discussion

Mental-related disorders, which impair students’ capacity to cope in academic settings, have been widely documented among university populations [1]. For example, a systematic review found that nearly one-third of university students in low- and middle-income countries experience burnout, often characterized by emotional exhaustion and cynicism, which may progress to psychiatric disorders, increased healthcare utilization, dependency, and disability [31]. To date, most interventions have focused on psychotherapeutic strategies and pharmacotherapy. However, critical evaluations have questioned the effectiveness of these interventions [32]. In this context, there is a growing call for alternative approaches to safeguard students’ well-being in higher education.

In line with this objective, this study assessed the frequency of three lifestyle factors, physical activity, chronotype, and disordered eating attitudes and behaviors, among undergraduate students. A secondary objective was to determine whether these factors are associated with subjective cognitive well-being, which is critical for effective functioning in academic settings. The study also examined the cohort’s sociodemographic profile, health history, frequency of these three lifestyle behaviors and their associations with subjective cognitive well-being. These aims are summarized below in tandem below within the existing literature.

First, regarding the sociodemographic profile, the majority of participants were undergraduate female students, reflecting broader national trends in higher education, where females are increasingly outnumber males [33], rather than being an artifact of recruitment. A significant proportion of participants reported that their parents had received formal education, consistent with national progress in literacy and educational attainment.

Second, this study has examined the health background of the cohort. Oman has been internationally recognized for its success in reducing communicable diseases and malnutrition. Despite this progress, the country has begun to witness a rise in lifestyle-related and non-communicable diseases, affecting even younger populations previously considered at low risk [34]. In this sample, only a small minority of participants reported having a chronic disease. Furthermore, some participants indicated that they had sought support for stress or mental health concerns. Very few participants reported experiencing adverse childhood events. Engagement in risky behavior was remarkably low: only a few participants reported smoking, and none reported alcohol consumption. This pattern may reflect sociocultural norms in which alcohol consumption is considered to offend social modesty.

Third, this study has explored the frequency of three lifestyle factors: eating attitudes, physical activity, and chronotype. According to the EAT-26 scale, participants in this study showed a tendency towards disordered eating attitudes and behaviors. This is slightly lower than the observed magnitude reported among school-aged youth in Oman [18], suggesting a reduced vulnerability with increasing age. However, the most critical assessment of the literature suggests substantial disparities in risk levels among tertiary education students [35]. Although, sex-specific differences in eating habits have been reported among young adults [36], no such association was observed in this study.

Fourth, levels of physical activity among young people vary globally and are influenced by sex, urbanization, and cultural factors. In the United States, 30–40% of young people engage in high levels of physical activity, although inactivity is more common among females. In Spain, approximately 40% report high activity levels, with urban youth being more active than their rural counterparts. In China, 25–35% report high activity levels, with notable regional and sex differences. In Saudi Arabia, 20–30% report high activity levels, and females tend to be less active than males. In Brazil, 35–45% report high activity levels, influenced in part by active commuting practices. In South Africa, activity levels vary widely due to socioeconomic factors, whereas in Iran, 20–30% report high activity levels, with cultural norms influencing participation [37]. In this study, more than half of participants self-identified as physically active. This proportion is higher than estimates for the general Omani population, in which 35% are reported to be sedentary [38]. This pattern may reflect greater health awareness among university students or differences in demographic characteristics compared with the general population.

Five, despite their importance for functioning, chronotypes have received limited attention among university students, with a few exceptions. Studies report that approximately 27% of students are morning types, 22% are evening types, and 50% are intermediate types [39]. In the present study, 22.5% of the students were classified as morning types, 34.1% as evening types, and 43.4% as neither type. These proportions are consistent with findings from Iraq, Lebanon, and the United Arab Emirates, where intermediate chronotypes are commonly observed among student populations [40,41]. Although, sex-specific differences in sleep habits have been reported among young adults [36], no association between sex and chronotype was observed in this study.

### 4.1. Associations of Lifestyle Behaviors with Subjective Cognitive Well-Being

Cognitive well-being is crucial for university students, as it supports their ability to learn and retain information. It also enhances essential academic skills, including attention, memory, and problem-solving. Moreover, adequate cognitive well-being is associated with better time management, adaptability, and decision-making skills, which are vital for independent living and future careers. Overall, cognitive well-being helps enable students to thrive academically, socially, and personally. Against this background, the central aim of this study was to identify lifestyle variables that are associated with cognitive well-being.

A forced-entry regression analysis revealed that chronotype was positively associated with physical activity (β = 0.06, *p* = 0.004), whereas disordered eating behavior was not significantly associated with physical activity. Moreover, physical activity was positively associated with cognitive function (β = 0.11, *p* < 0.001).

Although the regression analyses showed small effect sizes (β < 0.2) and the hypothesized SEM model had a suboptimal fit, the observed relationships were statistically significant. This suggests small yet statistically reliable associations between the variables and cognitive well-being. Moreover, the small effect sizes may indicate that additional, unmeasured factors also contribute to these associations. Previous research indicates that behaviors and mental health are multifactorial, shaped by multiple biological, psychological, and environmental influences, and that combined effects should be considered rather than examining each factor in isolation [7,42]. These findings suggest a network of interactions through which lifestyle factors interrelate; some may influence cognitive well-being indirectly by affecting other components of the lifestyle behaviors (physical activity, chronotype, and eating habits), warranting further investigation.

Physical activity levels (directly) and chronotypes (indirectly) may contribute, with small effects, to associations with cognitive well-being; however, when considered alongside additional factors and over longer periods, their collective influence may be greater. The observed association between physical activity and cognitive well-being aligns with a growing body of literature highlighting the role of exercise in reducing chronic stress [43]. In student populations, physical activity has been shown to reduce emotional distress and enhance academic coping. Furthermore, emerging evidence suggests that chronotype, although relatively underexplored, may be significantly associated with cognitive outcomes [44].

### 4.2. Implications

This study investigated a novel insight into how the three modifiable risk factors (physical activity, eating habits, and chronotypes) may simultaneously be associated with cognitive well-being. Findings from this study may provide a foundation for future research aimed at refining the proposed hypothetical model by incorporating additional factors. However, given the exploratory nature of this study, further research is needed to confirm and extend these findings and future longitudinal studies investigating the associations of physical activity, chronotypes, and eating habits with cognitive well-being across the undergraduate years may reveal a cumulative or interactive effects over an extended time or when combined with other factors. Similar investigations could also be conducted in non-academic settings or among different age groups.

Furthermore, findings from this study support the development of integrated interventions that promote physical activity and align academic routines with individual chronotypes such as the creation of low-cost pilot activities within school and university environments that encourage healthier lifestyle behaviors. Such approaches are sustainable and may yield both immediate and long-term benefits by reducing healthcare burden and associated costs.

### 4.3. Limitations

As with any cross-sectional survey, this study has several limitations that may affect the generalizability of its findings. First, the sample was drawn from a single public university, which may limit the applicability of the findings to students in private institutions or those studying abroad. Second, the sample was disproportionately female (74.3%), reflecting national trends, but introducing the possibility of sex-related bias. Third, the distribution of participants was skewed toward specific colleges, reducing the representativeness of the broader student population. Fourth, for QME questionnaire, no validated Arabic version is currently available. Fifth, this study was exploratory and lacked a pre-established theoretical baseline model; therefore, we evaluated a range of SEM models to identify the most appropriate model. Although significant associations were observed, the final model demonstrated suboptimal fit. The small effect size (β < 0.2) and suboptimal model fit may be partly attributed to the (non-normal distribution of the data, which can influence goodness-of-fit indices. These findings may also suggest a possibility of residual confounding factors or additional unmeasured factors that could cumulatively influence cognitive well-being over time, warranting examination in future studies. Furthermore, data collection relied on self-reported questionnaires, which are susceptible to recall bias, social desirability bias, and potential misinterpretation of items. It would have been ideal to determine how the participants defined chronic disease, an issue that warrants future investigation in future studies. Additionally, the use of subjective measures to assess perceived cognitive well-being may not fully capture its complexity when compared to objective neuropsychological assessments. Although the broader literature suggests that physical inactivity, poor sleep patterns, and disordered eating are precursors to students’ burnout, this study did not explore the variation in burnout or the presence of chronic stress in the present cohort. Future studies should address this issue. Finally, the cross-sectional design of the study precludes causal inference among the variables examined.

## 5. Conclusions

Amid growing concerns about the well-being of university students and the limited efficacy of existing interventions, this exploratory study examined whether modifiable lifestyle factors (physical activity, eating habits, and chronotype), are associated with cognitive well-being and may inform new avenues to supporting academic coping.

Among Omani undergraduates, physical activity was significantly associated with better subjective cognitive well-being; however, the effect size was small. Although disordered eating and chronotype were not directly associated with cognitive well-being, both were associated with physical activity, also with a small effect size, which in turn was directly associated with cognitive well-being. These findings underscore the interconnected nature of lifestyle behaviors and suggest that the small effects of multiple factors may cumulatively influence cognitive well-being over time or when considered alongside other variables. The modifiable nature of the examined risk factors, such as promoting physical activity and aligning academic routines with individual chronotypes, may offer a holistic approach to supporting student well-being and academic success. However, given the exploratory nature of this study, further research is needed to confirm and extend these findings.

## Figures and Tables

**Table 1:** Sociodemographic characteristics and risk factors of study participants (*n* = 408).

Variables	Subgroups	*n* (%)
**Age (Mean ± SD)**		408 (20.21 ± 1.566)
Sex	Male	105 (25.7%)
	Female	303 (74.3%)
Marital state	Married	11 (2.7%)
	Single	397 (97.3)
Father education	Illiterate	17 (4.2%)
	School	99 (24.7%)
	Institute	53 (13.2%)
	University	232 (57.9%)
	No response	7 (1.7%)
Mother education	Illiterate	44 (10.8%)
	School	161 (39.5%)
	University	201 (49.3%)
	No response	2 (0.5%)
Accommodation	Off-campus	187 (45.8%)
	On-campus	221 (54.2%)
Type of accommodation outside of campus	Individual	11 (6.1%)
	Shared	55 (30.2%)
	With the family	116 (63.7%)
	No response	226 (55.4%)
Academic level	Excellent	91 (22.3%)
	Very good	202 (49.5%)
	Good	113 (27.7%)
	Weak	2 (0.5%)
College	College of Agricultural and Marine Sciences	35 (8.6%)
	College of Arts and Social Sciences	27 (6.6%)
	College of Education	43 (10.5%)
	College of Engineering	40 (9.8%)
	College of Medicine and Health Sciences	131 (32.1%)
	College of Nursing	21 (5.1%)
	College of Science	62 (15.2%)
	Faculty of Economics and Science	28 (6.9%)
	Faculty of Law	19 (4.7%)
	No response	2 (0.5%)
Chronic disease	Yes	31 (7.6%)
	No	377 (92.4%)
Sought unified help for psychological distress	Yes	39 (9.6%)
	No	369 (90.4%)
History of adverse childhood experience	Yes	32 (7.8%)
	No	376 (92.2%)
Smoking	Yes	3 (0.7%)
	No	405 (99.3%)
Alcohol	Yes	0 (0%)
	No	408 (100%)

**Table 2:** Frequency of the lifestyle behaviors (physical activity, eating attitudes and chronotype) variables among students in tertiary education from Oman (*n* = 408).

Variables	Subgroups	*n* (%)
Eating Attitudes	Less than 20	292 (71.6)
	More than 20	116 (28.4)
Physical Activity	Inactive	193 (47.7)
	Active	212 (52.3)
Sleep/Wake chronotype	Morning-type	92 (22.5)
	Evening-type	139 (34.1)
	Neither	177 (43.4)

**Table 3:** Association between the lifestyle behaviors (physical activity, eating habits and chronotypes) and sociodemographic variables among tertiary education students in Oman (408).

Variables	Subgroups	Disordered Eating Behavior				Physical Activity *^$^*				Chronotype				
		Disordered *n* (%)	Normal *n* (%)	ES *	*p*	Inactive *n* (%)	Active *n* (%)	ES *	*p*	Morning *n* (%)	Neither *n* (%)	Evening *n* (%)	ES *	*p*
Sex	Male	27 (25.7)	78 (74.3)	0.04	0.53	38 (36.5)	66 (63.5)	0.13	0.01	22 (20.9)	43 (41.0)	40 (38.1)	0.05	0.56
	Female	89 (29.4)	214 (70.6)			155 (51.5)	146 (48.5)			70 (23.1)	134 (44.2)	99 (32.7)		
Marital status	Single	113 (28.5)	284 (71.5)	0.004	0.99	189 (47.8)	206 (52.2)	0.02	0.75	88 (22.2)	173 (43.6)	136 (34.2)	0.06	0.54
	Married	3 (27.3)	8 (72.7)			4 (40.0)	6 (60.0)			4 (36.4)	4 (36.4)	3 (27.2)		
Accommodation	On- campus	62 (28.1)	159 (71.9)	0.01	0.91	117 (53.2)	103 (46.8)	0.12	0.02	51 (23.1)	97 (43.9)	73 (33.0)	0.02	0.89
	Off-campus	54 (28.9)	133 (71.1)			76 (41.1)	109 (58.9)			41 (21.9)	80 (42.8)	66 (35.3)		
Academic level	Weak	1 (50)	1 (50)	0.01	0.92	1 (50)	1 (50)	0.06	0.72	1 (50)	1 (50)	0 (0)	0.17	0.04
	Good	31 (27.4)	82 (72.6)			58 (52.3)	53 (47.7)			26 (23)	46 (40.7)	41 (36.3)		
	Very Good	58 (28.7)	144 (71.3)			93 (46.3)	108 (53.7)			34 (16.8)	98 (48.5)	70 (34.7)		
	Excellent	26 (28.6)	65 (71.4)			41 (45.1)	50 (54.9)			31 (34.1)	32 (35.1)	28 (30.8)		
Chronic disease	Yes	14 (45.2)	17 (54.8)	0.11	0.04	19 (63.3)	11 (36.7)	0.09	0.88	6 (19.3)	14 (45.2)	11 (35.5)	0.02	0.91
	No	102 (27.1)	275 (72.9)			174 (46.4)	201 (53.6)			86 (22.8)	163 (43.2)	128 (34.0)		
Sought help for psychologic-al distress	Yes	20 (51.3)	19 (48.7)	0.17	0.002	17 (43.6)	22 (56.4)	0.03	0.62	5 (12.8)	16 (41.0)	18 (46.2)	0.09	0.16
	No	96 (26.0)	273 (74.0)			176 (48.1)	190 (51.9)			87 (23.6)	161 (43.6)	121 (32.8)		
History of adverse childhood experience	Yes	15 (46.9)	17 (53.1)	0.12	0.02	17 (53.1)	15 (46.9)	0.03	0.58	8 (25.0)	9 (28.1)	15 (46.9)	0.09	0.16
	No	101 (26.9)	275 (73.1)			176 (47.2)	197 (52.8)			84 (22.3)	168 (44.7)	124 (33.0)		
Smoking	Yes	1 (33.3)	2 (66.7)	0.01	0.99	2 (66.7)	1 (33.3)	0.03	0.61	0 (0.0)	0 (0.0)	3 (100)	0.12	0.05
	No	115 (28.4)	290 (71.6)			191 (47.5)	211 (52.5)			92 (22.7)	177 (43.7)	136 (33.6)		

* ES: Effect size. ***^$^*** No response from 3 students in Physical Activity.

**Table 4:** Independent regression analysis of the lifestyle behaviors (eating attitude, physical activity, and chronotypes) assessing their independent effects on physical activity and subjective cognitive well-being as dependent variables. EAT-26 (score); IPAQ (Active or inactive); chronotype (score).

	Equation 1			Equation 2		
	Dependent Variable: Physical Activity			Dependent Variable: Subjective Cognitive Function		
Variables	B (SE) *	β ^$^	*p*	B (SE) *	β ^$^	*p*
Chronotype	0.066 (0.022)	0.062	0.004			
Disordered eating behavior	0.014 (0.010)	0.013	0.16			
Physical activity				3.95 (1.91)	0.11	0.039

B (SE) *: coefficient (Standard error). β ^$^: Standardized coefficient.

## Data Availability

The de-anonymized raw dataset and the analytical script supporting the conclusions of this article will be made available by the authors on request.

## Supplementary Materials

The following supporting information can be downloaded at https://www.mdpi.com/article/10.3390/ijerph23010017/s1: Figure S1: Hypothetical model for direct pathways between eating attitude, physical activity, and chronotypes variables as predictors of subjective cognitive well-being (Standardized Root Mean Squared Residual (SRMR) = 0.29; Normed Fit Index (NFI) = 0.65; Comparative Fit Index (CFI) = 0.55; Tucker-Lewis Index (TLI) = 0.53; Root Mean Square Error of Approximation (RMSE) = 0.088, 90% CI: (0.085, 0.091); Table S1: Standardized effects of the lifestyle behaviors (eating attitude, physical activity, and chronotypes) on subjective cognitive well-being.

## Abbreviations

The following abbreviations are used in this manuscript:

EAT-26	26-item Eating Attitudes Test
IPAQ-SF	International Physical Activity Questionnaire
MEQ-5	The 5-item Morningness–Eveningness Questionnaire
