# Supplementary material for: Lifestyle Behaviors and Cognitive Well-Being: A Cross-Sectional Study Exploring the Role of Lifestyle Factors Among Omani University Students
Source: Int J Environ Res Public Health. 2025 Dec 22;23(1):17. doi: 10.3390/ijerph23010017 (PMC12841314; doi:10.3390/ijerph23010017)
Supplement: Supplementary file 1 [file ijerph-23-00017-s001.zip › ijerph-3976841-supplementary.docx]

**Supplementary Materials**

**
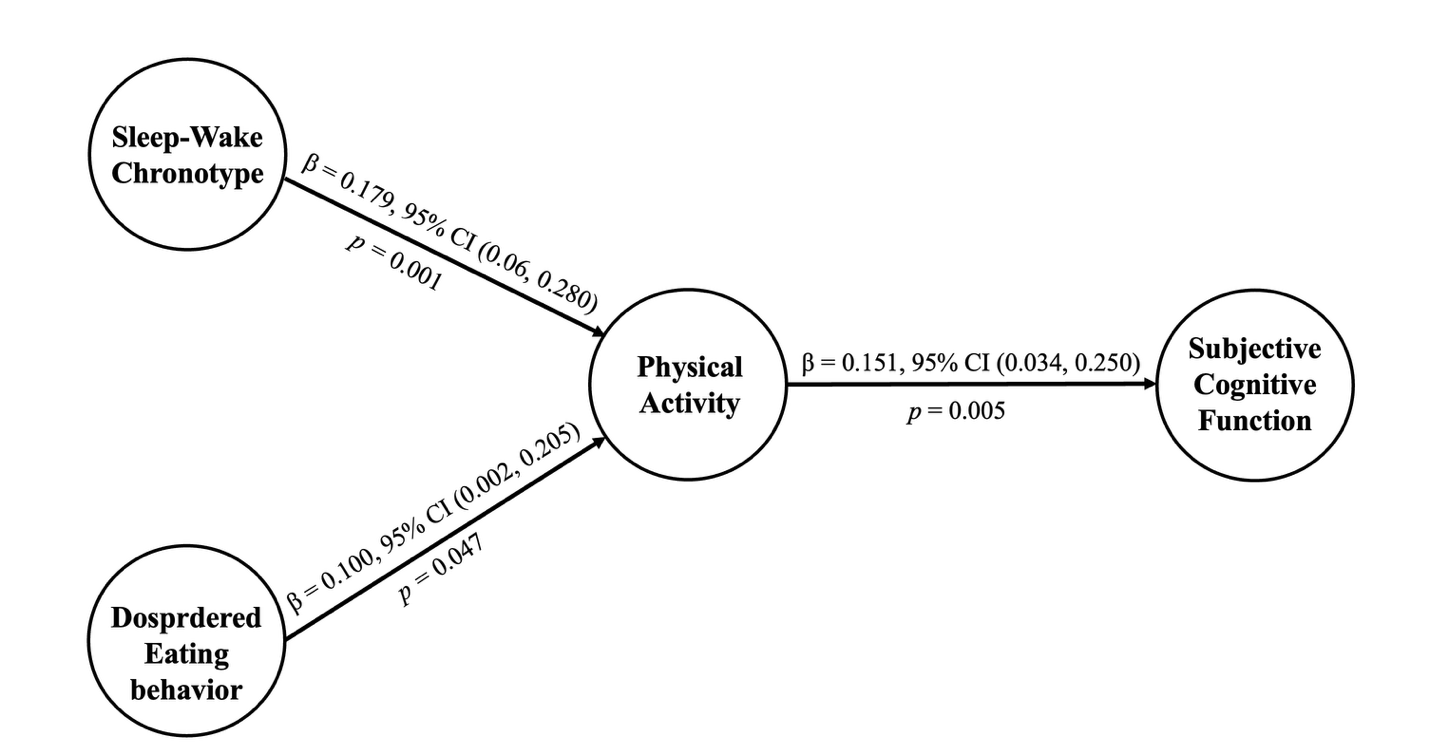
Figure S1.** Hypothetical model for direct pathways between eating attitude, physical activity, and chronotypes variables as predictors of subjective cognitive well-being (Standardized Root Mean Squared Residual (SRMR) = 0.29; Normed Fit Index (NFI) = 0.65; Comparative Fit Index (CFI) = 0.55; Tucker-Lewis Index (TLI) = 0.53; Root Mean Square Error of Approximation (RMSE) = 0.088, 90% CI: (0.085, 0.091).

**Table S1.** Standardized effects of the lifestyle behaviors (eating attitude, physical activity, and chronotypes) on subjective cognitive well-being.

| **Pathway** | **β (95% CI)** | ***p*** | **FDR^*^** |  |
| --- | --- | --- | --- | --- |
| Physical activity $->$subjective cognitive well-being | 0.151 (0.034, 0.250) | 0.005 | 0.017 |  |
| Chronotypes $->$ Subjective cognitive well-being | 0.027 (0.004, 0.058) | 0.046 | 0.033 |  |
| Disordered eating behavior $->$ Subjective cognitive well-being | 0.015 (-0.001, 0.037) | 0.115 | 0.05 |  |

* **FDR: False Discovery Rate (Benjamini–Hochberg)**
